# Pain characterization and response to palliative care in dogs with naturally-occurring appendicular osteosarcoma: An open label clinical trial

**DOI:** 10.1371/journal.pone.0207200

**Published:** 2018-12-06

**Authors:** Beatriz P. Monteiro, Louis-Philippe de Lorimier, Maxim Moreau, Guy Beauchamp, Jeffrey Blair, Bertrand Lussier, Jean-Pierre Pelletier, Eric Troncy

**Affiliations:** 1 GREPAQ (Groupe de recherche en pharmacologie animale du Québec), Department of biomedical sciences, Faculty of Veterinary Medicine, Université de Montréal, Saint-Hyacinthe, Quebec, Canada; 2 Centre Vétérinaire Rive-Sud, Brossard, Quebec, Canada; 3 Osteoarthritis Research Unit, University of Montreal Hospital Research Centre (CRCHUM), Montreal, Quebec, Canada; 4 Vétoquinol SA, Global–Le Groupe Vétoquinol, Magny-Vernois, France; Colorado State University, UNITED STATES

## Abstract

This study aimed to characterize bone cancer pain (quantitative sensory testing (QST), stance asymmetry index, actimetry, scores of pain and quality of life (QoL)) in dogs with appendicular osteosarcoma (OSA), and to evaluate a stepwise palliative analgesic treatment.

The pain profile of thirteen client-owned dogs with OSA was compared with seven healthy dogs. Dogs with OSA were then enrolled in a prospective, open-label, clinical trial. Outcome measures included: primary and secondary mechanical thresholds (MT), conditioned pain modulation (CPM), stance asymmetry index, actimetry (most and least active periods), visual analog scales and QoL. After baseline assessments, stepwise treatment comprised orally administered cimicoxib (2 mg/kg q 24h), amitriptyline (1–1.5 mg/kg q 24h) and gabapentin (10 mg/kg q 8h); re-evaluations were performed after 14 (D14), 21 (D21) and 28 (D28) days, respectively. Statistics used mixed linear models (α = 5%; one-sided). Centralized nociceptive sensitivity (primary and secondary MT, and dynamic allodynia) was recorded in OSA dogs. Healthy dogs had responsive CPM, but CPM was deficient in OSA dogs. Construct validity was observed for the QST protocol. Asymmetry index was significantly present in OSA dogs. The CPM improved significantly at D14. When compared with baseline (log mean ± SD: 4.1 ± 0.04), most active actimetry significantly improved at D14 (4.3 ± 0.04), D21 and D28 (4.2 ± 0.04 for both). When compared with baseline, least active actimetry significantly decreased after treatment at all time-points indicating improvement in night-time restlessness. No other significant treatment effect was observed. Except for tactile threshold and actimetry, all outcomes worsened when gabapentin was added to cimicoxib-amitriptyline. Dogs with bone cancer are affected by widespread somatosensory sensitivity characterized by peripheral and central sensitization and have a deficient inhibitory system. This severe pain is mostly refractory to palliative analgesic treatment, and the latter was only detected by specific and sensitive outcomes.

## Introduction

Cancer is the number one cause of mortality in dogs [1], and pain is a common clinical feature leading to stress, suffering, and low scores of quality of life (QoL) [2,3]. Osteosarcoma (OSA) is an aggressive and invasive neoplasm of the skeletal system that causes both osteolytic and proliferative changes. It is the most commonly diagnosed primary bone tumor in dogs [4,5] and is generally associated with a poor long term prognosis [6]. Bone cancer pain is characterized by peripheral and central sensitization with both nociceptive and neuropathic components [7]. In people, it is described as dull in character and constant in presentation, and bone remodeling results in severe spontaneous pain. Breakthrough pain is also a clinical feature characterized by episodes of extreme pain that can occur spontaneously or be induced by movement. These clinical signs tend to progress over time and patients become severely affected with altered function and QoL [7,8]. The actual incidence and characteristics of cancer pain in dogs remain unknown [9]. Nevertheless, given that dogs frequently present with advanced-stage cancer at initial evaluation and have similar cancer biology when compared with humans [10], it is reasonable to presume that canine patients experience a similar pain profile [2]. Research aiming to further enhance our understanding of cancer pain in animals is clearly warranted. A recent survey of veterinarians in the United Kingdom revealed that 87% agreed or strongly agreed that cancer pain is underdiagnosed, and 66% disagreed or strongly disagreed with the statement “pain associated with cancer is easy to treat” [11]. Under-diagnosis and under-treatment of cancer pain is also a reality in human patients, particularly when neuropathic pain is involved [12].

The World Health Organization proposes a 3-stepwise palliative pharmacologic approach for the management of cancer pain [13]. Patients with mild pain are administered NSAIDs, and patients with moderate to severe pain are administered opioids in combination with adjuvant analgesics. In veterinary medicine, oral administration of opioids does not seem to produce significant anti-nociception [14]. Opioids are controlled drugs with limited availability worldwide and potential for abuse. For these reasons, veterinarians may be reluctant to prescribe opioids for outpatients.

Previous studies have evaluated the efficacy of intravenous administration of bisphosphonates [15], and intrathecal administration of substance-P saporin [16], and resiniferatoxin [17]. Although efficacy was observed with these treatments, they can be relatively costly or are not readily available or require technical skills that make their use extremely limited. Therefore, this study proposed a stepwise palliative analgesic treatment including orally administered medications that are available and of low cost, namely cimicoxib, an NSAID [18], amitriptyline, a tricyclic antidepressant drug [19], and gabapentin, an inhibitor of voltage-dependent calcium channels [20]. None of these analgesics, to the best of the authors’ knowledge, has been evaluated in dogs with OSA-related pain. The nature and intensity of OSA-related pain has not been characterized, nor has the response to a stepwise palliative analgesic treatment. This lack of knowledge impairs proper recognition and treatment of pain in dogs with bone cancer [2,3].

The study objectives were to characterize OSA-related pain in dogs by evaluating their somatosensory system using different quantitative sensory testing (QST) applicable in clinical conditions, scores of pain and QoL, assessment of the level of activity and sleep disturbance, and to test the efficacy of a non-opioid stepwise palliative analgesic treatment.

The study hypotheses were that the somatosensory profile of OSA compared to healthy dogs could be characterized, with OSA dogs presenting peripheral and central sensitization, decreased activity, sleep disturbance and poor QoL scores, and that these outcome measures would significantly improve with treatment, indicating analgesic response after palliative analgesic therapy when compared with baseline values.

## Materials and methods

The study was approved by the *Comité d’Éthique de l’Utilisation des Animaux* (CÉUA Rech-1806), registered on the American Veterinary Medical Association (AVMA) Animal Health Studies Database (#AAHSD-000362 08/01/2016 to 04/17/2018) and is reported according to the ARRIVE guidelines. The individual in this manuscript has given written informed consent to publish these case details.

### Animals and experimental protocol

The study was divided into two phases. First, healthy (n = 7) and OSA (n = 13) dogs were compared using a standardized QST protocol and an asymmetry index (AI) measurement. Second, OSA dogs included in a prospective open-label clinical trial were evaluated using the same outcome measures, in addition to actimetry as well as pain and QoL scores.

Healthy client-owned dogs from the staff and students of the Faculty of Veterinary Medicine, Université de Montréal were selected for the first phase. Dogs were considered healthy if they had a normal physical examination and no history of trauma or any orthopedic or systemic disease. They also had to be good-tempered in order to accept manipulation and younger than 3 years of age. For selection of client-owned OSA dogs, the study was advertised to all referring veterinarians within a 100 km radius of the Centre Vétérinaire Rive-Sud (CVRS), where the clinical trial was conducted from August 2016 to April 2018. Inclusion criteria for OSA dogs were confirmed diagnosis of appendicular OSA by a board-certified oncologist using cytology or histopathology, or a very high suspicion of OSA based on signalment, history and radiographs (location and appearance of the bone lesion), and evidence of cancer-related pain. Dogs had to be good-tempered and conventional cancer treatments such as surgery, radiation, chemotherapy or bisphosphonates were not a viable option for the dog or had been declined by the owner. Dogs could not be receiving any analgesic at the time of inclusion. If the dog was being administered analgesics during screening, a wash-out period of 10 days for NSAIDs or steroids, and 2 days for centrally-acting analgesics such as tramadol or acetaminophen, was required. Dogs that presented with metastatic disease but were in good health could be included.

Dogs were excluded if they presented with severe concomitant disease, a survival time prognosis of less than one month, history of gastrointestinal disease, uncontrolled endocrine disease, history of NSAID intolerance, azotemia with chronic kidney disease IRIS stage 2 or higher [21], or ALT greater than three times the upper reference limit. Dogs were not included if there was any question regarding the owner’s compliance with treatment, knowledge of the dog`s normal behavior or intellectual capacity.

Eligible dogs were included after a thorough discussion with the owner and an informed consent form had been signed. During the study, dogs were excluded if any adverse effects requiring intervention developed or if any medication outside the protocol was administered.

### Outcome measures

#### Standardized QST protocol

Evaluation of the somatosensory profile of dogs was performed using a QST protocol that was specifically designed for this study (dx.doi.org/10.17504/protocols.io.uwzexf6 and dx.doi.org/10.17504/protocols.io.uw4exgw).

**Fig 1:**
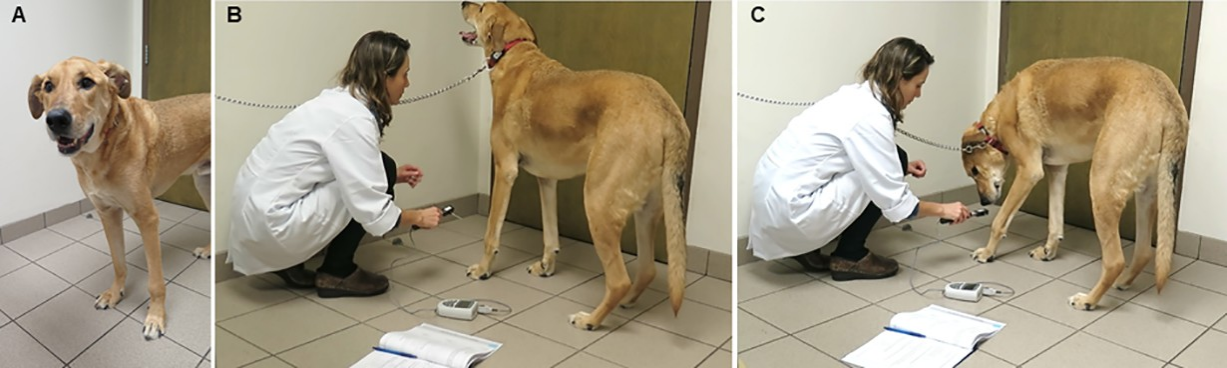
Primary tactile threshold test. (A) A 12-year-old neutered male mixed breed dog with OSA of the left distal radius. (B) Before the stimulus, the dog is bearing weight on the affected limb and looking around. (C) Behavioral response to the stimulus: the dog is looking at the stimulated area and withdrawing the weight from the limb.

Static QST, which typically assesses the sensory thresholds to or the rating of a single stimulus, included primary tactile threshold, and primary and secondary mechanical nociceptive thresholds (MTs). Potential primary tactile threshold was evaluated using an electronic von Frey anesthesiometer (with Rigid tip of 0.7 mm^2^ surface, 28G, IITC Life Sciences Inc, Woodland Hills, CA, USA) with a 1000g internal load cell [22]. Briefly, gradually increasing pressure was applied perpendicular to the skin, dorsal to the right metacarpus (between metacarpal bones III and IV) in healthy dogs and perpendicular to the tumor in OSA dogs, until a behavior response indicative of a conscious perception of the stimulus was observed (*e*.*g*. paw withdrawal, reaching for the device, vocalization, *etc*.) (Fig 1). The peak force was recorded, and a safety cut-off was defined at 500g. Mechanical nociceptive threshold was evaluated using a pressure algometer (Digital Force Gauge Series 3, Model M3-2, Mark-10, Copiague, NY, USA) with a 10N internal load cell connected to a blunt “W” shaped metal tip (primary MT) or a sharp pointed metal tip (secondary MT). Tests of MT were performed similarly to the primary tactile threshold tests and a safety cut-off was defined at 10N. Triplicate measurements of primary tactile threshold and primary MT were done with a 10-second interval between each test and the average was calculated. Tests of secondary hyperalgesia were performed over the paravertebral muscles (right and left sides) immediately caudal to the 11^th^ and 13^th^ vertebra in both healthy and OSA dogs [23]. Triplicate measurements were done at these four locations with 1-minute intervals between them, and the mean of the resulting 12 values was calculated for each time-point.

**Fig 2:**
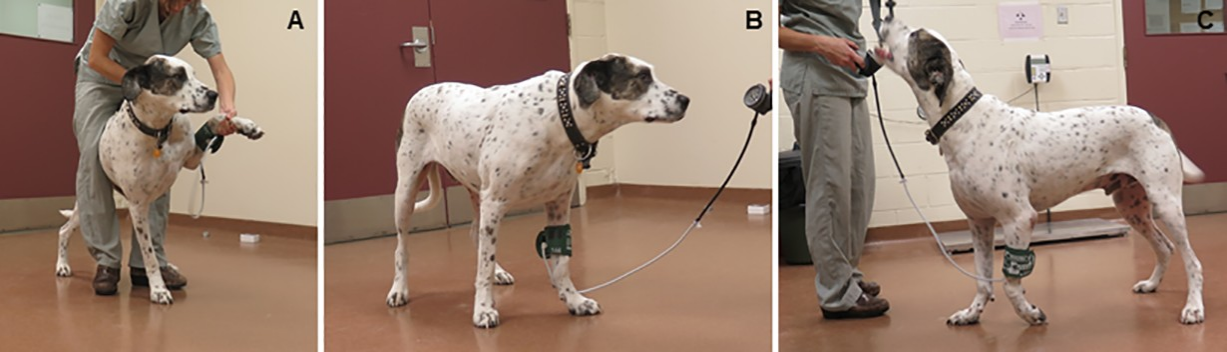
Conditioning stimulus (ischemic noxious model) for conditioned pain modulation (CPM) test in a healthy dog. (A) The dog’s left thoracic limb is gently lifted and pressed/massaged from the paw towards the elbow or stifle joint. Then, a pressure cuff is placed around the mid-radius and inflated up to 200 mmHg. (B) The dog is encouraged to walk around the room for two minutes. (C) Note the left non-weight-bearing limb demonstrating the discomfort caused by the ischemia (conditioning stimulus).

Dynamic QST, which assesses the response to a number of stimuli [24–27], has gained increasing attention because it offers the opportunity to probe the central processing of incoming nociceptive signals [25,26]. In this study, dynamic QST included brush-evoked allodynia, and conditioned pain modulation (CPM). Brush-evoked allodynia was evaluated using a purposely-designed device with a soft bristle that was gently brushed along the hair growth up to three times or until a behavior response was observed. Dogs were defined as being affected by brush-evoked allodynia if a response was observed, and later evaluated as a proportion of positive responders. Referring to the phenomenon of “pain inhibits pain”, CPM is the behavioral correlate of diffuse noxious inhibitory control (DNIC), where the presence of a second noxious stimulus (*i*.*e*., conditioning stimulus) decreases the pain perception from an initial noxious stimulus (*i*.*e*., test stimulus) [25,26]. The test-retest stimulus were MT and the conditioning stimulus was ischemic pain (Fig 2), as adapted from protocols in humans [28]. In healthy dogs, the conditioning stimulus was done in the left thoracic limb, whereas in OSA dogs, it was done in the limb diagonal to the affected limb (*i*.*e*., if the tumor was in the left thoracic limb, the conditioning stimulus was done in the right pelvic limb). For the conditioning stimulus, the limb of the dog was gently lifted and pressed/massaged from the paw towards the elbow or stifle joint. Then, a pressure cuff was placed around the mid-radius or mid-tibia, inflated up to 200 mmHg, and the dog was encouraged to walk around the room for two minutes. Once the two minutes were complete, the retest-stimulus (*i*.*e*., MT) was immediately performed in quadruplicate (twice while the cuff was inflated and twice after cuff deflation) with 10-second intervals between each measurement. The mean of these four values was used as the MT post-conditioning stimulus. Data from the primary MT were used as the MT (pre-conditioning) test stimulus. The difference between retest and test MT, *i*.*e*., post- minus pre-conditioning stimulus was calculated (Delta CPM) and dogs were classified (Functional CPM rate) as having a functional (Delta CPM ≥ 0) or dysfunctional DNIC system (Delta CPM < 0) [29]. The QST protocol was always completed in the same room and with the same staff to whom the dogs had been accustomed since the initial visit. The somatosensory tests were performed while the dogs were in a standing position by one of two evaluators (BPM or LPdL). The latter was taken into consideration during statistical analyses. Dogs were allowed to rest between tests and were given food rewards once the QST protocol was completed.

#### Scores of pain and QoL

Pain was independently evaluated by the veterinary oncologist (LPdL) and the owner using a 10 cm visual analog scale (VAS) in which “0” corresponds to “no pain” and “10” corresponds to the “worst imaginable pain” [30]. At each visit, the oncologist scored the VAS after the physical examination and the owner scored the VAS according to what he/she believed the dog had experienced on average in the last five days. The oncologist and the owners were not aware of each other’s VAS scoring. Scores of QoL were done using a French language questionnaire specifically designed for the study (dx.doi.org/10.17504/protocols.io.uw5exg6 and dx.doi.org/10.17504/protocols.io.uw6exhe) according to previously published literature on the subject [31–34]. The QoL questionnaire included 19 items pertaining to three main domains: happiness (1 to 4), physical functioning (5 to 12), and quality of life (13 to 19). A 20^th^ item was available to be added and scored if the owner believed that there was a specific behavior relevant to the dogs that had not been addressed in the questionnaire. In addition, the owners could choose not to score up to eight items they believed were irrelevant in their dog’s case. Unscored items were then crossed out for the subsequent visits. The owners were also asked to choose the item they believed was the most relevant within each domain. Items could be scored using a 4-point Likert scale adapted for each item; thus, higher scores indicate higher impact from the disease in the dog’s QoL. A final score was calculated as the percentage of the given scores divided by the maximum possible score according to the total number of scored items for each dog. At each time-point, the owners were systematically asked how the dog was doing since the last visit and were left alone in the room without further conversation. They were given the VAS and QoL questionnaire to be completed. Except for the baseline visits, owners were also given the previously scored QoL questionnaire since dependent interviewing has been shown to increase treatment effect sizes [35]. The same owner completed both the VAS and QoL questionnaire at all time-points.

#### Objective outcomes: Actimetry and asymmetry index

Actimetry was assessed using an accelerometry-based activity sensor attached to the collar throughout the entire study period (Actiwatch-64; Bio-Lynx Scientific Equipment, Inc., Montreal, QC, Canada). Actimetry (intensity of motor activity; no unit; from 0 to infinite) epoch was two minutes. Once the data were downloaded, the 1-hour (30 counts) intensity was summated. Then, the ten most and least active 1-hour periods were selected for each time-point of assessment. Finally, the average of these ten 1-hour periods was calculated, so that each dog had a mean actimetry value for its least and most active periods for each time-point. The most active periods reflected the level of activity while the dog was active. The least active periods reflected the periods in which the dog was sleeping and are a measure of sleep disturbance (*i*.*e*., the lesser the activity during the least active period, the better the sleep) [36].

Static weight bearing data were collected while dogs were standing over a pressure-sensitive mat (PetSafe Stance Analyzer, Model 300–2509, Vet therapy–Kruuse A/S, Langeskow, Denmark) with one paw in each of four quadrants. A series of eight data (proportion of body weight per paw) was collected during one minute at each time-point and its mean was calculated. Asymmetry index was calculated according to a previously published formula [37], which accounts for the asymmetry in weight-bearing between two contralateral limbs. Thus, the greater the asymmetry, the higher the AI. In healthy dogs, AI was calculated for the thoracic limbs. In OSA dogs, AI was calculated for the affected limbs (*i*.*e*., thoracic or pelvic limbs depending on the anatomical location of the tumor).

#### Monitoring of adverse effects

Complete blood cell count, serum chemistry profile and urinalysis were performed at each time-point, except for Baseline 2. The owners were thoroughly educated as to which adverse effects to monitor and were instructed to contact the researchers if they were observed.

#### Treatments and assessment time-points

In the first phase, healthy and OSA dogs were evaluated twice for each outcome measure. Evaluation of the healthy dogs was done on the same day (morning and afternoon), whereas for the OSA dogs, Baseline 1 and 2 were separated with an interval of 2 to 4 days in order to evaluate the repeatability of baseline assessments. After Baseline 2, all OSA dogs were prescribed treatment level 1 and were re-evaluated after 14, 21 and 28 days (D14, D21, and D28, respectively). At each re-evaluation, the treatment was changed to a higher level in a stepwise approach if at least one of the following conditions was observed: QoL scores did not decrease from the previous visit or were > 40%, or VAS scores from owners or the oncologist did not decrease or were > 4.0. Level 1 treatment comprised cimicoxib (Cimalgex chewable 8, 30 and 80mg tables, Vetoquinol S.A, Lure, France) administered at 2 mg/kg orally every 24 hours. Level 2 treatment comprised cimicoxib (same dosage) in addition to amitriptyline (Apo-amitriptyline 10 and 25mg, Apotex, Toronto, ON, Canada) administered at 1–1.5 mg/kg orally every 24 hours. Level 3 treatment comprised cimicoxib (same dosage), amitriptyline (same dosage), in addition to gabapentin (Apo-gabapentin 100 and 300mg, Apotex) administered at 8–10 mg/kg orally every 8 hours. Upon study completion, a long-term follow up was done by telephone interview.

### Statistical analyses

Data were analyzed using SAS (version 9.3, SAS Institute, Inc., Cary, NC, USA). Data were tested for normality using the Shapiro-Wilk test. For comparisons between healthy and OSA dogs, data were analyzed using independent *t*-tests, Mann-Whitney or Fisher’s exact tests where appropriate. Pre- and post-conditioning stimulus MTs for CPM testing were compared with paired *t*-test. Repeatability of baseline measurements and the association between the VAS scores from owners and the oncologist or between the scores of VAS and QoL were calculated using intraclass correlation coefficient (ICC), kappa coefficient or Pearson’s correlation coefficient. Results from ICC or kappa/Pearson statistics were interpreted using the Altman’s classification (0.81–1.00 excellent; 0.61–0.80 good; 0.41–0.6 moderate; 0.21–0.4 fair and < 0.2 poor) [38]. The average of both baseline measurements was calculated and used for subsequent comparisons over time. The effect of time and treatment on numerical variables was analyzed using a linear mixed model and analyses were one-tailed with regard to treatment efficacy hypothesis. Time was considered a fixed effect and dog a random effect for treatment. The QST evaluator, sex of the owner, anatomical location of the tumor (thoracic *versus* pelvic) and body weight were added to the model as covariates where appropriate. Data from actimetry were log-transformed for analyses to normalize distribution. The effect of time and treatment on categorical variables was analyzed using the Cochran-Mantel-Haenszel test for repeated measures. Adjustments for multiple comparisons were done using the Benjamini-Hochberg sequential adjustment procedure. The level of statistical significance was set at 5%.

## Results

### Inclusion data

Seven healthy dogs were included. Twenty-eight OSA dogs were screened for eligibility and 13 met the inclusion criteria. Of these, 11 dogs completed the study. Individual characteristics of included OSA dogs are presented in Table 1; this data is consistent with previous studies on canine OSA. Fifteen dogs were not included in the study for the following reasons: being treated with other analgesics and compliance with the wash-out period required for inclusion would be too painful (n = 10), aggressiveness (n = 1), no signs of pain (n = 1), treatment with prednisone due to hyperadrenocorticism (n = 1), euthanasia before first visit due to rapid progression of disease (n = 1); unavailability of owner to be present at all required visits (n = 1).

**Table 1:** Individual characteristics of dogs with naturally-occurring osteosarcoma included in the study.

Breed	Sex	Age (years)	Body weight (kg)	Anatomical location of the tumor
**Golden Retriever**	Female	7.7	44.2	Left distal radius
**Cane Corso**	Male	7.8	64.5	Left distal radius
**Great Dane**	Male	4.7	70.8	Right distal tibia
**Labrador Retriever X Great Dane**	Male	8.9	54.9	Left distal radius
**Newfoundland**	Female	5.9	64.4	Right distal radius
**Rottweiler**	Female	9.0	41.1	Left proximal humerus
**Siberian Husky**	Male	11.5	22.4	Right proximal humerus
**Rottweiler**	Male	5.7	48.7	Right proximal tibia
**Mastiff X Great Dane**	Male	9.1	48.6	Right distal radius
**Great Dane**	Female	6.0	64.2	Right proximal humerus
**Siberian Husky**	Female	10.3	23.2	Right distal femur
**Great Dane**	Male	7.7	53	Right distal radius
**Great Dane**	Female	4.2	52.5	Right distal radius
	**Mean ± SD**	**7.6 ± 2.2**	**50.1 ± 14.7**	

Two dogs were excluded after baseline evaluations (one due to development possible of adverse effects and another due to euthanasia because of rapid progression of the disease). Baseline data from these two dogs were included in the data analysis. All data from one dog at D28 were excluded because the owner had not been able to administer the medications on the previous three days. Data for the QST protocol for one dog at D28 were not collected because the dog became aggressive during manipulation. Actimetry data for one dog and AI for another dog at D14, 21 and 28 could not be collected due to technical issues. The remaining data were all included in the analyses.

In the first phase, all QST tests and AI were significantly different between healthy and OSA dogs, except for primary tactile threshold (Table 2).

**Table 2:** Quantitative sensory testing (QST) and asymmetry index measured by static weight bearing in healthy dogs (n = 7) and dogs with naturally-occurring appendicular osteosarcoma (OSA) (n = 13).

Outcome measure	Status	Mean ± SD	Frequency	*p-*value
**QST** **Primary tactile threshold** (grams) Lower value means sensitization	Healthy	297.6 ± 185.2	-	0.079
	OSA	198.4 ± 65.4	-	
**Primary mechanical threshold** (Newtons) Lower value means sensitization	Healthy	8.9 ± 0.6	-	0.012^a^
	OSA	6.6 ± 2.2	-	
**Secondary mechanical threshold** (Newtons) Lower value means sensitization	Healthy	9.8 ± 0.5	-	0.001^a^
	OSA	5.7 ± 2.5	-	
**Brush-evoked allodynia** (% of positive responders) High value means sensitization	Healthy	-	0	0.026^a^
	OSA	-	53.8	
**Delta CPM** (Newtons) Must be positive in normal conditions	Healthy	0.6 ± 0.6	-	0.014^a^
	OSA	-0.5 ± 1.5	-	
**Functional CPM rate** (%) Close to 100 in normal conditions	Healthy	-	85.7	0.035^a^
	OSA	-	38.5	
**Asymmetry index** (%) Close to 0 in normal conditions	Healthy	6 ± 2	-	0.000^a^
	OSA	30 ± 14	-	

CPM: conditioned pain modulation.

^a^Indicates *p*-values that are rejecting the null-hypothesis of the test (absence of difference between groups).

### Repeatability of baseline measurements

Repeatability for healthy dogs was good to excellent for primary tactile threshold, secondary MT, brush and functional CPM rate, and fair for primary MT, delta CPM and AI. In OSA dogs, ICC for primary tactile threshold, and primary and secondary MTs was 0.33, 0.50 and 0.91, respectively. The kappa coefficient for functional CPM and brush-evoked allodynia was 0.36 and 0.09, respectively. The ICC for the owners’ scores of VAS and QoL were 0.58 and 0.87, respectively, and 0.90 for the oncologist’s VAS score. The ICC for AI was 0.78.

Data from OSA dogs before and after treatment are reported in Table 3. All OSA dogs received the three levels of treatment.

**Table 3:** Outcome measures of dogs with naturally-occurring osteosarcoma (n = 13) before and after 14, 21 and 28 days of a stepwise palliative analgesic treatment in which they received cimicoxib (2 mg/kg PO q 24h), cimicoxib + amitriptyline (1–1.5 mg/kg PO q24h) and cimicoxib + amitriptyline + gabapentin (10 mg/kg PO q 8h), respectively.

Outcome measure	Baseline	Day 14	Day 21	Day 28
**Primary tactile threshold** (grams)	178.1 ± 28.6	152.2 ± 30.2	190.25 ± 30.0	231.0 ± 33.1
**Primary mechanical threshold** (Newtons)	5.8 ± 0.6	4.7 ± 0.7	5.2 ± 0.6	4.7 ± 0.7
**Secondary mechanical threshold** (Newtons)	5.2 ± 0.8	4.7 ± 0.8	5.2 ± 0.8	5.1 ± 0.8
**Brush-evoked allodynia** (% of positive responders)	46.2	45.5	27.3	44.5
**Delta CPM** (Newtons)	-0.5 ± 0.5	1.2 ± 0.6	0.9 ± 0.6	0.6 ± 0.7
**Functional CPM rate** (%)	38.5	90.9^a^	81.8	75.0
**Oncologist’s VAS** (no unit) Higher value means more pain	4.3 ± 0.4	4.3 ± 0.4	4.5 ± 0.4	5.0 ± 0.5
**Owner’s VAS** (no unit) Higher value means more pain	4.5 ± 0.4	5.2 ± 0.4	4.8 ± 0.4	5.0 ± 0.4
**Quality of life** (%) Higher value means less QoL	51.3 ± 5.3	57.1 ± 5.5	56.0 ± 5.6	57.5 ± 5.5
**Quality of life (most relevant item)** (%) Higher value means less QoL	65.3 ± 28.1	66.7 ± 29.8	60.6 ± 25.0	73.3 *±* 21.1
**Log-transformed actimetry: least active** (no unit)	2.3 ± 0.08	1.8 ± 0.1^a^	2.0 ± 0.1^a^	1.9 ± 0.1^a^
**Log-transformed actimetry: most active** (no unit)	4.1 ± 0.04	4.3 ± 0.1^a^	4.2 ± 0.1^a^	4.2 ± 0.1^a^
**Actimetry: least active** (no unit)^b^ Lower value means less restlessness	224.4 ± 118.3	69.8 ± 19.8^a^	99.3 ± 38.2^a^	83.7 ± 34.0^a^
**Actimetry: most active** (no unit)^b^ Higher value means more mobility	11039.7 ± 2964.9	20273.6 ± 6912.6^a^	16430.2 ± 3914.5^a^	14740.5 ± 4768.8^a^
**Asymmetry index** (%)	35.5 ± 4.2	40.8 ± 4.5	37.7 ± 4.5	40.9 ± 4.6

Data are presented as least square mean ± SEM unless otherwise stated.

^a^Indicates a significant difference when compared with baseline.

^b^Descriptive data presented as mean ± SD.

### Standardized QST protocol (Table 3)

Primary tactile threshold and primary MT did not change across time *(p =* 0.15 for both) and were not affected by anatomical location *(p =* 0.31 and 0.12, respectively) or the evaluator *(p =* 0.35 and 0.17, respectively). Secondary MT did not change across time *(p =* 0.1) and was not affected by the evaluator *(p =* 0.38) or the anatomical location (*p =* 0.42).

Response to brush-evoked allodynia did not change across time *(p =* 0.36). When MTs pre- and post-conditioning stimulus for CPM testing were compared at each time point, a difference between them was observed at D14 *(p =* 0.03), but not at other time-points. Delta CPM did not change across time (*p =* 0.07) and was not affected by the evaluator (*p* = 0.55) or the anatomical location (*p* = 0.71). Compared to baseline, functional CPM rate increased significantly at D14 (*p* = 0.016), and slightly decreased subsequently (Table 3). Except for primary tactile threshold and least active actimetry, all D28 outcomes worsened when gabapentin was added to cimicoxib-amitriptyline at D21 (Table 3).

### Scores of pain and QoL (Table 3)

Oncologist VAS score did not change across time *(*p = 0.37) and was not affected by the anatomical location of the tumor (*p* = 0.56). Owner VAS and QoL scores did not change across time (*p* = 0.41 and 0.47, respectively). Owner VAS score was higher in dogs whose tumors were in the pelvic limbs (least square mean ± SEM: 6.0 ± 0.6) when compared with the thoracic limbs (3.7 ± 0.3) (*p* = 0.006), and in dogs whose owners were women (n = 8, 5.8 ± 0.4) when compared with men (n = 5, 3.9 ± 0.5) *(p =* 0.01). Owner QoL score was not affected by anatomical location (*p* = 0.09), but women (65.1% ± 5.5) scored higher than men owners (45.7% ± 7.0) (*p* = 0.04). At first assessment, the most relevant items were “Willingness to play” (for 53.9% of respondents) in the “Happiness” domain, “Mobility (walking, trotting or running)” (38.5%) in the “Physical functioning” domain, and “My dog’s overall quality of life” (38.5%) in the “Quality of life” domain. Only three items, in the latter domain, were never used by respondents, namely “I am concerned about my dog’s general appearance”, “My dog seems dull or depressed, not alert”, and “My dog shakes or trembles”. When considering only the most relevant item throughout the study, namely “My dog’s overall quality of life”, which was the 19^th^ item in the questionnaire, it is interesting to note that the owners attributed higher score (65.3%, see Table 3) to their evaluation. Oncologist’ and owner’s VAS score correlations were moderate and good (Pearson’s correlation coefficient = 0.44 and 0.77; p = 0.13 and p = 0.01) at baseline and D14, respectively, but poor (D21: 0.10; p = 0.78) and fair (D28: 0.32; p = 0.37), subsequently. Owner VAS and QoL scores were better correlated: Pearson’s correlation coefficient = 0.89, 0.85, 0.59 and 0.72, at baseline, D14, D21 and D28 (p = 0.0001; 0.0009; 0.06 and 0.02), respectively.

### Objective outcomes: Actimetry and asymmetry index (Table 3)

Compared to baseline, the least active actimetry decreased significantly at D14, D21 and D28 (*p* < 0.0001 for all). It was not affected by body weight (*p =* 0.48) or anatomical location (*p* = 0.94). The most active actimetry increased at D14 (*p* < 0.0001), D21 (*p* < 0.0001) and D28 (*p* = 0.0003). It was not affected by body weight (*p* = 0.25) or anatomical location (*p* = 0.0545). The effect of anatomical location was nearly significant, and it is interesting to note that dogs with tumors in the thoracic limbs (least square mean (log actimetry) ± SEM: 4.1 ± 0.03) were less active than those with tumors in the pelvic limbs (4.3 ± 0.05). Asymmetry index remained unaltered across time (*p* = 0.45) and was not affected by the evaluator (*p* = 0.96). Asymmetry index was higher in dogs with tumors in the pelvic (least square mean ± SEM: 48.2% ± 6.4) than in the thoracic limbs (29.3% ± 3.8) (*p* = 0.03).

### Survival time

Data on the only surviving dog (male) at the time of this report were not included in the survival time analysis. Median (range) survival time was 43 (28–208) days since study inclusion. Survival time was weakly and negatively correlated with owner VAS and QoL scores from the last visit (Pearson’s correlation coefficient = -0.04 and -0.13, respectively).

### Monitoring of adverse effects

One male dog was withdrawn after three days of treatment with cimicoxib (level 1) due to development of liquid diarrhea with hematochezia and melena. Treatment was stopped, and the dog recovered within 24h. Another dog developed progressive depression, anorexia and polyuria on D24. This dog was euthanized on D28 after data collection. Apart from these two dogs, no other clinically significant adverse effect was observed, except for increase in renal parameters over time for some dogs. Therefore, a linear mixed model for repeated measures was performed with values from creatinine, urea and urine specific gravity to qualify potential renal alteration. There was no significant change over time for creatinine or urine specific gravity (*p* > 0.11). Compared to baseline, urea values increased at D14 (*p* = 0.026), D21 (*p* = 0.003) and D28 (*p* = 0.001), but abnormal values (outside normal range [2.5–9.6 mmol/L]) were present on 7 occasions, for 5 dogs. Other observed abnormalities included mild anemia (n = 2 occasions), increase in serum alkaline phosphatase (n = 1 occasion) and mild to moderate neutrophilia (n = 2 occasions). Overall, cimicoxib appeared to be well tolerated in this senior, cancer-bearing dog population.

## Discussion

The profile of dogs with primary bone cancer was characterized, demonstrating widespread somatosensory sensitivity and dysfunction of the descending inhibitory nociceptive modulation (DNIC). This translated to a high degree of biomechanical alteration (as assessed by AI). Subjective pain assessment was on average about 50% of VAS and QoL scales. Construct validity of the proposed QST protocol was observed, based on the premise that if the outcome could actually measure sensory sensitivity, values between healthy and OSA dogs would be different. The responsiveness to analgesic palliative treatment was difficult to objectify: the responsiveness was present on actimetry and dynamic QST (indicating improvement in DNIC), more pronounced with cimicoxib alone, and did not change either static QST, or AI, or subjective assessments (VAS and QoL). The expected synergic addition of amitriptyline and gabapentin did not provide more analgesia, and could have even worsened the dogs’ pain.

In the first phase, all static and dynamic QSTs were different between healthy and OSA dogs, except for primary tactile threshold. In order to further explore this potential lack of difference, we analyzed data from the OSA dogs (198.4 g ± 65.4, mean ± SD) in comparison with historical control of healthy dogs (n = 21; 403.4 g ± 135.6) (*p* < 0.0001). Thus, the absence of difference between healthy and OSA dogs for primary tactile threshold is plausibly related to a type-II statistical error due to the small sample size (n = 7) of healthy dogs, especially if one considers their large SD observed for this outcome (Table 2). Large variability in primary tactile threshold was also observed in recent studies comparing healthy and osteoarthritic dogs [22], and cats [39].

Dynamic QST brings another dimension to the neurophysiological phenotyping of animal pain. Brush-evoked allodynia QST was easy to complete and presented specific response (none of the healthy dogs responded to brushing), but moderate sensitivity with 54% of OSA dogs being positive responders (but the rate of sensitized OSA dogs could not be estimated). Although we cannot know the perceptual quality of this stimulation in animals, it is believed that the response to brushing is associated with dynamic mechanical allodynia. Indeed, in people with chronic pain, touch pleasantness elicited by brushing is decreased when compared with healthy individuals [40]. To the best of our knowledge, this is the first report of CPM in healthy dogs in comparison to dogs with chronic pain. Due to the lack of such literature in dogs, we opted to explore CPM effects in three different ways: by evaluating the difference between the pre- and post-conditioning stimulus MTs, by comparing the delta CPM between groups and with a binary classification of (dys)functional CPM rate. Regardless of the approach, descending inhibitory nociceptive modulation in the OSA dogs of our study was clearly affected in comparison with healthy dogs, concurring with a well-established finding in people with chronic pain [29,41].

The AI was very different between healthy and OSA dogs, demonstrating a quasi-absence of imbalance in healthy dogs, and a 30% on average, contralateral report to the unaffected limb in OSA dogs. A previous study established an interval between 15.7–19.5% in which dogs with AI above this interval were affected by cranial cruciate ligament rupture differentiating them from healthy dogs [37]. It is interesting to note that dogs with OSA had nearly twice as much asymmetry than orthopedically-affected dogs, emphasizing the severe clinical presentation in the former population.

The ICC for QST assessments varied largely from poor to good [38]. Interpretation of these results is based on the assumption that the measures are stable [42], which might not be the case in OSA patients. In addition, variability of the data might be related to stress, learning [43], or the evaluator’s technique and interpretation. For example, previous research has shown that learning confounds algometric assessment in normal dogs since their thresholds decreased over time, with dogs anticipating the stimulus and reacting at lower thresholds [43]. Similar results with no replicate effect of QST measures have been reported in previous studies with dogs [44,45].

In the second phase, with the exception of actimetry and dynamic QST, outcome measures (namely, static QST, AI, and subjective pain/QoL assessment) did not statistically change with treatment. Different explanations can be considered:

1. Nature of bone cancer pain: The efficacy of analgesic therapies might depend on the specific population of sensory neurons that innervate bone [7]. For example, primary afferent sensory neurons from the bone are restricted to specific A-delta and C-fibers [46]. In addition, recent research identified that canine OSA cells express and secrete nerve growth factor, endothelin-1 and prostaglandin E_2_ which potentially participate in malignant bone pain [9]. Finally, microfracture can result from progressive osteolysis by activated osteoclasts [7]. The sensitivity (responsiveness to treatment) of most assessment outcomes of this study could be too low, or the analgesic effect of the tested treatment could be too small, in view of such established chronic pain mechanisms.
2. Degree of neuropathic pain: In OSA, neuropathic pain can be caused by direct nerve damage or compression from the tumor, and by an active and pathological sprouting and neuroma formation by nerve fibers that innervate the skeleton [7]. A systematic review of studies in people reported a liberal estimate of the prevalence of neuropathic pain in patients with cancer to be 39% [12], and the pharmacotherapy of neuropathic pain is challenging and frequently unsatisfactory in people [47].
3. Rapid disease progression: In people, it is well known that the prevalence of pain increases with disease progression at a rate of 70–90% in patients with advanced disease [47]. The pain prevalence appears similar in canine cancer (75.2%) [33]. Considering that most dogs in this study had locally advanced OSA, and that the median survival time was 43 days from study inclusion, it could be argued that the proposed analgesic approach was not enough to counteract the pain and evolution of disease.
4. Drugs and dosage protocol: The rationale of the proposed protocol attempted to counteract peripheral and central mechanisms of pain by using centrally- and peripherally-acting analgesics. Cimicoxib is a cyclooxygenase (COX)-2 selective inhibitor (‘coxib’) licensed in Europe for the long-term management of pain and inflammation associated with osteoarthritis [18]. Amitriptyline is a tricyclic antidepressant drug that inhibits the reuptake of serotonin and norepinephrine in the central nervous system, and is therefore expected to reinforce the descending inhibitory nociceptive modulation [19]. Gabapentin is an anticonvulsant drug with analgesic properties mediated *via* blockade of certain voltage-dependent calcium channels, and is therefore expected to inhibit the ascending nociceptive transmission [20]. Amitriptyline and gabapentin are the first line of treatment for neuropathic pain in humans [48,49]. It is possible that the dosage and treatment duration were not adequate to observe efficacy. Furthermore, it might be plausible that using orally administered analgesics is not enough to control bone cancer pain.
5. Small sample size: Calculation of sample size and power analysis could not be performed since there was no available data from the literature regarding pain in OSA dogs. A trend of effects was clearly observed for some of the outcome measures, such as primary tactile threshold, CPM, and brush-evoked allodynia (with the addition of amitriptyline); however, these were not statistically significant, due to a type II statistical error.

Regarding sensitive criteria able to detect a treatment efficacy, dynamic QSTs, and specifically CPM, are attractive. It was possible to include their use during clinical examination after a short period of learning. The baseline functional CPM rate was largely deficient in OSA dogs. It improved after two weeks of cimicoxib treatment and was maintained with the addition of amitriptyline and gabapentin, which supports the hypothesis of a central analgesic effect of the coxib drug. This reinforcement of a deficient descending inhibitory nociceptive modulation was more expected with the antidepressant drug, amitriptyline [29]. Indeed, at D21, the functional CPM rate in OSA dogs showed a trend to be improved, and the percentage of positive responders to brush-evoked allodynia to be decreased. Further validation and investigation of CPM in clinical trials may clarify the role of CPM in canine pain medicine.

Most and least active periods were affected by treatment. Activity monitoring is a non-invasive, valid and widely used outcome to assess spontaneous activity in dogs with chronic pain [50], and their response to treatment [51]. In this study, it was hypothesized that this pain syndrome would be associated with discomfort during rest (translated to increased ‘low’ activity) and during active motion (translated to decreased ‘high’ activity). Subsequently, the analgesic treatment, if efficient, would lead to a lower actimetry during least active periods (or, improvement in the sleep quality) [36], and to a higher actimetry during most active periods. Both results were observed to be significant in the present study, herein suggesting an improvement in sleep quality and more ease in active movement of the OSA dogs treated with cimicoxib. The addition of complementary analgesics did not result in additional improvement in these actimetry outcomes.

Scores of both VAS and QoL fluctuated over time with no improvement. One limitation of this study was the use of a non-validated clinical metrology instrument to assess QoL. However, based on a recent systematic review [52,53], and the fact that there was no validated French language instrument, we designed the QoL questionnaire based on all available information [31–34]. It remains unknown whether the lack of improvement in QoL was real, or due to the lack of validity of the tool itself. Considering that some objective outcomes responded to analgesic treatment, the second hypothesis is more plausible. Finally, this study contributes to the psychometric validation of the QoL questionnaire, as some criteria appear to be excluded, and other presented some trend to respond to treatment. The deterioration in the most relevant (for owner) item throughout the study of the QoL scale from D21 to D28 evaluation is interesting, as the owners quoted highly the alteration for “My dog’s overall quality of life”, in addition to using it often in the scale, and is suggestive of a deterioration in the dogs’ condition with the addition of gabapentin. The fact this item was the last one of the QoL questionnaire makes it attractive since it came after the owner thought about the previous items and it is a global QoL assessment criterion.

Another limitation of this study is the intrinsic bias from scores of pain and QoL for the owners and the clinician during post-treatment evaluations. Nevertheless, with the absence of any effect, including a placebo effect, it seems that observer bias was not relevant in this study. Furthermore, it would have been ethically unacceptable to include a placebo-control group in this clinical trial.

The results observed in this prospective open-label clinical trial highlight an intriguing point about systematic use of multimodal therapies. At D28, after one week of gabapentin addition, it is obvious that, with the exception of primary tactile threshold and least active actimetry, and although not significant, all outcome measures deteriorated, when compared with D21. The decrease in delta CPM at D28 is significant compared to D14 and is suggestive of a deterioration in the descending inhibitory nociceptive modulation. Such pharmacological association is based on benefits observed in preclinical models [54,55], but it is frequent for preclinical promises not to translate to similar clinical benefits [56], and such observation warrants caution before recommending systematic multimodal analgesia protocols for canine cancer pain. It is possible that these findings were related with progression of the disease. Furthermore, the fact that most outcome measures did not improve in the present study raises an ethical concern regarding the palliative treatment of dogs with OSA. Further studies are unquestionably necessary to answer to this question.

The study results suggest that the aggravated bottom-up sensitization (widespread somatosensory sensitization) and the impaired endogenous descending pain inhibition (CPM) characterize canine patients with bone cancer pain similarly to people with severe chronic painful conditions [7,8,28]. This is the first report to characterize the nature of bone cancer pain in dogs and it suggests that neurophysiological pain assessment using standardized static and mostly dynamic QSTs are attractive to promote pain mechanisms-based analgesic therapy in veterinary patients.

## Supporting information

S1 Appendix Database. (XLSX)
